# Wound of the Knee Joint

**Published:** 1870-04

**Authors:** J. F. Miner


### ART. II.—*Wound of the Knee Joint.* By J. F. Miner, M. D.

Wound of the *knee joint* is one of the most common, and also, one of the most severe and dangerous injuries. The expressions of the older authors lead us to believe that most injuries extending into the knee joint will prove so serious as to make primary amputation necessary, or at least more safe than secondary amputation which is later required if the patient survive the fever and constitutional disease commonly induced. The acute synovitis which so generally follows wounds opening into the knee joint, the liability of purulent absorption and death from *pyœmia,* and the probability that if no more serious consequences follow, anchylosis will be the result, prove how serious and dangerous all wounds which reach to the synovial cavity of the knee may be regarded. Recent advances in surgery have demonstrated how exsections may often take the place of amputations, and how many cases of synovitis may be safely and successfully treated by the mildest measures. The successes which have attended operations upon joints, have also operated to abate our fears in compound dislocations, and in penetrating wounds or other injuries complicating the larger joints, but the experience of modern surgeons will be found to correspond in many respects with that of the older authors. It is true that we sometimes see punctured wounds extending into the larger joints productive of no serious symptoms or consequences; see compound dislocations terminate as favorably as simple, being followed by no untoward symptoms, but such is by no means the general rule, and the opinions of the earlier authors, will be sustained in a great degree, by recent observers. It is not probable that surgeons at the present time would consider primary amputation indicated in penetrating wound of the knee joint, since now it is with great reluctance and hesitation that we advise amputation; we should not, perhaps, entertain at all, their measures of treatment, but could scarcely abate from their opinions of the severity and danger of such injury. The literature of the profession upon this subject leaves the young surgeon to doubt and hesitancy as to the safest and best methods of procedure and the questions which have been so long and extensively discussed are still, in his mind, at least, unsettled.

It has been my misfortune to see nearly every case of incised or punctured wound extending into the knee joint which has fallen under my observation, followed by very serious consequences, though I have observed similar injuries, in nearly all the other joints, terminate favorably. This leads me to infer that a wider experience may furnish other instances of complete recovery from wounds extending into the knee joint, rather than that injury of the knee is greatly more dangerous than the shoulder, elbow, hip, ankle, &c. These injuries are certainly common, but the experience of any one observer furnishes a quite inadequate basis for conclusion. The idea formerly so prevalent that air admitted to serous membranes and to synovial surfaces, is very dangerous, almost always provoking acute inflamation, has been modified somewhat by the experience of recent observers, by the success attending operations upon abdominal tumors, and by the result of exsections and other operations upon joints, so that the profession is better prepared to expect favorable termination after injury of joints than formerly. Judging from my own experience alone, I should abate nothing from the severity and danger formerly attached to such injury, and should differ only in the mode of treatment. Amputation is more our last resource than formerly; sufficient courage could not be obtained to make amputation after injury of the knee joint from incised or punctured wound; on cases only of great injury to the articular surfaces and ends of the bones, could such a procedure be considered.

My thoughts have been turned to consideration of these accidents by cases which have recently fallen under my care, the brief histories of which are instructive. The first was on the person of a carpenter, who struck his axe through the integuments upon the inner side of the left leg, opening the knee joint, and dividing a small artery. Pressure appeared to control the hemmorhage, but, he was obliged to ride three miles into the country, to his boarding house. During the night he suffered great pain, and, in the morning I was invited to visit him. Blood had escaped from the artery and had infiltrated in considerable quantity between the muscles and under the fascia, and it was found necessary to enlarge the opening and ligate the artery. Violent inflamation supervened in the joint, and the constitutional symptoms of purulent absorption soon manifested themselves. He died three months after the injury from pyæmia.

The second case was caused by the draw shave of a cooper dividing the tissues in front of the joint, making a large broad incision completely into, and fully exposing the joint surfaces. The wound was carefully approximated, leg laid over a pillow, and water dressings applied. During the first three weeks no unpleasant symptoms had appeared. He now moved from his bed and attempted use of the leg. A few days later, the symptoms of synovitis commenced, the joint surfaces suppurated, and at the end of six months when last seen, he was walking with a crutch, and had anchylosis of the joint.

The third case is more recent, being now under treatment. Mr. L., carriage maker, struck his axe into the right knee, severing an artery which bled very profusely. Compress and tight bandages controlled the hemmorhage so that he was brought to my office for first dressing. The axe was found to have penetrated the synovial cavity of the knee joint, and the bleeding vessel had been compressed superficially so as to direct the current of blood into the joint cavity, from which the synovial fluid had been evacuated. After tying the vessel, the coagulated blood was pressed as much as possible from the joint and the patient ordered rest, anodynes, and water dressings. This patient is very sick, now suffering from traumatic delirium in addition to all the symptoms of acute synovitis. Pus and a greatly increased quantity of synovial fluid escape constantly from the wound. The leg is swollen, and very painful, and all the evidences of great constitutional disturbance are present. A little improvement for the last few days encourages the expectation that he will recover; but of this there is still much uncertainty.

Going back in line of personal experience and I could relate cases where wounds penetrating other joints were followed by no untoward symptoms, and where recovery was as rapid and complete as if only simple integument had been divided. It does not always follow that wounds extending into joints are attended by these serious consequences, but it is believed that the greatest risk is incurred and that as a rule, with but comparatively few exceptious, serious ill effects do follow.

It might be interesting to enquire into the causes of these dangerous and often fatal symptoms, but we should soon be liable to find ourselves without definite and well settled facts, wandering off into the regions of fancy and conjecture. It seems now settled that amputation through the knee is safer and better than through the thigh, showing, at least, that there is nothing in the anatomical character or physiological action of the synovial surface which prevents union or greatly exposes to the absorption of pus. It has been said that the articular surfaces are of low vitality and take on reparative action slowly and uncertainly, but this objection is hardly sustained by our observations of its vitality and activity when inflamed, or the readiness with which it unites with other tissues under favorable circumstances.

The influence of atmospheric air upon the articular surfaces of joints, has always been the source of fear and dread with surgeons; to how great an extent this is well grounded, it is difficult to determine, nor is it my purpose to consider to any great extent how this may be. It seems probable that the admission of air does have an important influence upon the joint surfaces and character of the secretions furnished; other causes may also act to stimulate inflammatory action, and prevent rapid recovery after such disease has once been established. To avoid admission of air, operations upon the knee are sometimes made under water, while with a view to prevent other unfavorable influences, various antiseptic and refrigerant applications have been used.

The *treatment* of these accidents have been greatly simplified within the past few years, though it may be noticed that Erichsen in the last edition of his surgery, speaks of the treatment thus:

"In fact, the three great principles of treatment in the early stages of wounds of joints, consists of the exclusion of air, perfect rest, and the continuous application of cold. In this way inflammatory action may be prevented, and union of the wound effected; but in the majority of cases the inflammation that is set up in the joint causes so abundant a secretion of synovial fluid, that the dressing becomes loosened by the tension and outward pressure of the accumulated fluid which escapes from under it. If the preventive means of arresting inflammation fail, and the joint swells, becoming red, hot and throbbing, with much constitutional irritation, the line of treatment must be changed, and means should be taken to limit the inflammatory action. This is best done by free application of leeches over the joint, hot fomentations, and the internal administration of calomel and opium about four times in the day. This remedy possesses a more decidedly controlling influence over traumatic arthritis than any other with which I am acquainted."

Careful review of these few sentences would occupy too much space and cannot now be presented to our readers; suffice to say, the sound reasoning and correctness of all this, is by no means established. It is all well to give directions to exclude the air, but its admission or exclusion is a matter over which the surgeon has little control. As to the application of "continuous cold," before the redness, heat, swelling and throbbing appear, or the free application of leeches, hot fomentation and internal administration of calomel and opium after, much differences of opinion would prevail. It is certainly as easy to do injury by our therapeutic measures as good, and it requires great discrimination to be able to reject the objectional modes of treatment and select such as, at least, shall do no harm. Some of the indications are very plain, viz: Closing the wound, preventing and abating inflammation, and relieving pain. That mercury, leeching, blistering, &c., &c., could produce any favorable effect, may as well be left for the result of individual experience and observation.

We naturally and rightfully expect from our remedies favorable effects; and often too readily accept the progress of disappearing disease as the benign influence of medicine.

